# A Study on the Oxygen Permeability Behavior of Nanoclay in a Polypropylene/Nanoclay Nanocomposite by Biaxial Stretching

**DOI:** 10.3390/polym13162760

**Published:** 2021-08-17

**Authors:** Bich-Nam Jung, Hyun-Wook Jung, Dong-Ho Kang, Gi-Hong Kim, Jin-Kie Shim

**Affiliations:** 1 Korea Packaging Center, Korea Institute of Industrial Technology, Bucheon 14449, Korea; jbn5666@kitech.re.kr (B.-N.J.); kangppp@kitech.re.kr (D.-H.K.); kakamate@kitech.re.kr (G.-H.K.); 2 Department of Chemical and Biological Engineering, Korea University, Seoul 02841, Korea; hwjung@grtrkr.korea.ac.kr

**Keywords:** polymer-matrix composites (PMCs), nanoclays, biaxial stretching

## Abstract

Polypropylene (PP) has poor oxygen barrier properties, therefore it is manufactured in a multi-layer structure with other plastics and metals, and has been widely used as a packaging material in various industries from food and beverage to pharmaceuticals. However, multi-layered packaging materials are generally low in recyclability and cause serious environmental pollution, therefore we have faced the challenge of improving the oxygen barrier performance as a uni-material. In this work, PP/nanoclay nanocomposites were prepared at nanoclay contents ranging from 0.8 to 6.4 wt% by the biaxial stretching method, performed through a sequential stretching method. It was observed that, as the draw ratio increased, the behavior of the agglomerates of the nanoclay located in the PP matrix changed and the nanoclay was dispersed along the second stretching direction. Oxygen barrier properties of PP/nanoclay nanocomposites are clearly improved due to this dispersion effect. As the biaxial stretching ratio and the content of nanoclay increased, the oxygen permeability value of the PP/nanoclay nanocomposite decreased to 43.5 cc·mm/m^2^·day·atm, which was reduced by about 64% compared to PP. Moreover, even when the relative humidity was increased from 0% to 90%, the oxygen permeability values remained almost the same without quality deterioration. Besides these properties, we also found that the mechanical and thermal properties were also improved. The biaxially-stretched PP/nanoclay nanocomposite fabricated in this study is a potential candidate for the replacement of the multi-layered packaging material used in the packaging fields.

## 1. Introduction

Plastic materials have been used as a packaging material in the past due to their low price, good mechanical strength, and easy processability. However, in recent years, plastic waste has been a major problem due to several factors. First, the consumption of packaged food, fresh food, and food delivery has increased at the highest reported growth rate for plastic packaging waste during the lockdown period, due to the coronavirus disease 2019 (COVID-19) pandemic [1]. Additionally, the e-commerce market continues to grow, and e-commerce services tend to use more packaging materials than conventional retail due to delivery security [2]. Although the use of plastic packaging materials lasts less than a week—due to durability, which is the greatest asset of plastic—it has resulted in an environmental waste problem [3]. To solve this problem, it is necessary to develop a material that does not consist of two or more polymer components, while having the same performance as currently used packaging material.

Polypropylene (PP) is a good candidate for packaging material due to its advantages, such as having a low cost, a high mechanical strength, excellent moisture barrier properties, easy processability, and a high recyclability [4]. However, PP has poor oxygen barrier properties, therefore its use in the packaging fields requiring oxygen barrier performance is limited. Many researchers have been trying to improve the oxygen barrier performance of PP for several decades. Among them, the manufacturing of PP-based composite materials using different additives and methods such as coating (protein [5,6], polyelectrolyte [7], silicate [8,9,10], and graphene oxide [11]) or stretching has been effective.

Improving the oxygen barrier performance of PP-based composite materials using different additives has been the most active study reported. PP-based composite materials can be classified as a carbon-based additive, such as graphene [12,13], derived from natural products such as cellulose [14,15] and nanoclay [16,17]. In this work, cheap and abundant nanoclay derived from natural products was selected as an additive material to be filled in the PP-based nanocomposite. Nanoclay (montmorillonite (MMT)), which has a structure with a thickness of approximately 1 nm and a length of several hundred nm, is a material with a very large aspect ratio and is stacked in several layers. One of the smectite groups, MMT, is a 2:1 nanoclay with two tetrahedral silica sheets sandwiched between a central octahedral sheet of alumina [18]. Peeling the stacked nanoclay into individual nanoclay layers is an important key in improving oxygen barrier performance, and the individual nanoclay layers can be uniformly dispersed in the PP matrix to form a tortuous path that inhibits gas diffusion. Recently, techniques have been applied to fabricate polymer/nanoclay nanocomposites by dispersing nanoclays into polymers by methods such as solution casting using biomimetic [19], in-situ polymerization [20], layer by layer coating [21], and melt mixing [22,23,24].

The biaxial stretching process, which manufactures films by controlling the arrangement and crystallinity of the PP chain, is also one of the processes used to improve oxygen barrier properties. In addition, the control of the free volume of the amorphous region of the polymers is also a factor affecting the gas permeability, and a previous study improving the gas barrier performance by reducing the free volume through the stretching process of the polymer has been reported [25]. In the case of commercial mass production, in the past, production was only possible using the sequential stretching method with unbalanced draw ratios, but now, it is possible to simultaneously stretch products in two perpendicular directions at the same time without limitation. Moreover, it has been reported that the samples prepared with unbalanced draw ratios under the sequential stretching process displayed better oxygen barrier properties compared with samples prepared with the same draw ratio [26].

To the best of our knowledge, there is no study on the oxygen barrier performance of a PP nanocomposite containing nanoclay developed through the biaxial stretching process. Based on previous research, to maximize the oxygen barrier performance of PP/nanoclay nanocomposites, a sequential stretching process and unbalanced draw ratio conditions were adopted in this study. In this study, PP/nanoclay nanocomposites were prepared by increasing the content of nanoclay in PP from 0.8 to 6.4 wt%, and the dispersibility of nanoclay was investigated through biaxial stretching. Through various analyses, it was confirmed that thermal stability, tensile strength, Young’s modulus, and oxygen barrier properties were enhanced due to the improved dispersibility of the nanoclay.

## 2. Materials and Methods

### 2.1. Materials

PP resin T3410 (melt index of 7 g/10 min at 230 °C and a melting point of 134.4 ± 0.7 °C), manufactured by LG Chem (Seoul, South Korea), was used as the matrix material. The PP was produced into microsized particles by cryogrinding. The nanoclay (Cloisite 20A) was used as purchased from BYK Additives and Instruments (Wesel, Germany). For nanoclay, the quaternary ammonium used as an organic modifier was dimethyl di(hydrogenated tallow), in which the majority of the double bonds were hydrogenated. The modifier concentration of nanoclay was 95 meq/100 g clay.

### 2.2. Preparation of Biaxially Stretched PP/Nanoclay Nanocomposite Films

The surface of microsized PP was modified by plasma treatment. The modification of microsized PP was carried out under controlled conditions with 10 min exposure time, gas of N_2_, and 50 W of plasma power using a radio frequency plasma generator (FEMTO Version E of 13.56 MHz, Diener electronic, Ebhausen, Germany). All the PP/nanoclay nanocomposites (0.8, 2.0, 3.4, 5.0, 6.4 wt%) were manufactured using an internal mixer (W50 Plastograph, Brabender GmbH & Co KG, Duisburg, Germany) equipped by a twin-screw at a temperature of 190 °C and a 80 rpm rotation speed. The surface-modified microsized PP and nanoclay were first premixed for 1 min and then melt-mixed for 5 min in internal mixer.

**Figure 1:**
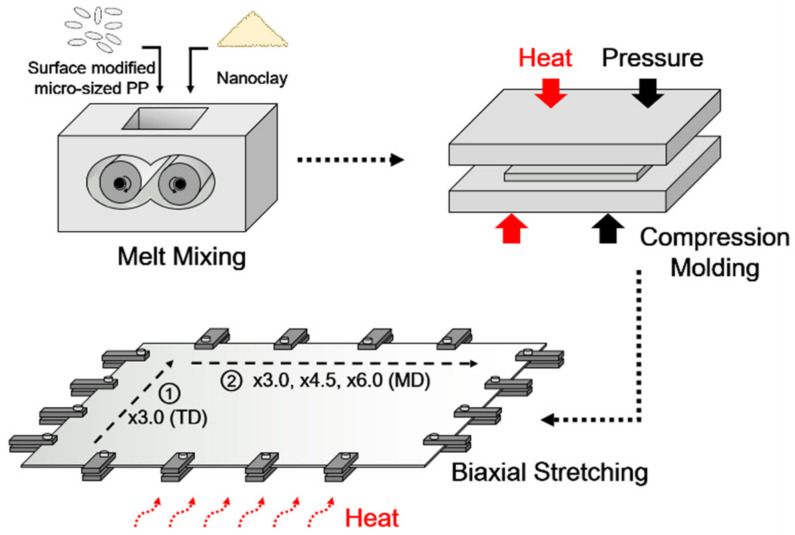
Schematic illustration of biaxially stretched PP/nanoclay nanocomposite film preparation.

The bulk-shaped nanocomposites samples were ground and dried at 105 °C for 12 h, and the nanocomposite granules were then compression molded at 190 °C using a hot press system (QM900A, QMESYS, Uiwang, South Korea). After compression molding, the mass change of nanoclay in nanocomposite sheets were analyzed using thermogravimetric analyzer (TGA) (Q500, TA Instruments, New Castle, DE, USA). The PP/nanoclay nanocomposite sheets were biaxially oriented using a biaxial stretcher (Biaxial Drawing Machine, Plus Ko-lab, South Korea) at 130 °C (semi-molten state) with a draw speed of 100 mm/s, and draw ratios were 3.0 × 3.0 (×9.0), 3.0 × 4.5 (×13.5), and 3.0 × 6.0 (×18.0). Biaxial stretching was sequentially performed. Here, the sheet was stretched in the transverse direction (TD) to a target draw ratio of ×3.0 while being constrained in the machine direction (MD). After a pause of 1 s that was imposed by the instrument, the sheet was stretched in the MD to the target draw ratio (×3.0 or ×4.5 or ×6.0) while being constrained in the TD. A schematic illustration of stretched PP/nanoclay nanocomposite films is shown in Figure 1.

### 2.3. Characterization

The low magnification morphology of the PP/nanoclay nanocomposites was observed with field emission scanning electron microscopy (FE-SEM, SU8020, Hitachi, Tokyo, Japan). The film samples were cryomicrotomed at −100 °C through the thickness direction using the cryomicrotome (LEICA ULTRACUT UC7, Leica, Wetzlar, Germany) equipped with a diamond knife. All the samples were coated with a Pt/Pd alloy using an ion sputter (E-1045, Hitachi, Tokyo, Japan).

Transmission emission microscopy (TEM, HT7700, Hitachi, Tokyo, Japan) was used to observe PP/nanoclay nanocomposites at high magnification. TEM samples, approximately 80 nm thick, were prepared from the PP/nanoclay nanocomposite that was extracted from the film using the cryomicrotome.

The phase structural analyses of the PP/nanoclay nanocomposites were performed by X-ray diffraction (XRD, D8 ADVANCE, Bruker, Billerica, MA, USA) with CuKα radiation (*λ* = 0.154 nm). The diffraction spectra were obtained at a 2θ range of 1–10, and the interlayer distance (d_001_) was calculated using the Bragg’s equation:

(1) nλ=2dsinθ

where λ is the wavelength, θ is the diffraction angle, and d is the interlayer distance between nanoclays.

The thermal stability and residual amounts of nanoclay in all PP/nanoclay nanocomposites were studied by TGA at N_2_ atmosphere from room temperature to 600 °C at a heating rate of 10 °C/min.

Tensile properties were determined with film samples using a universal testing machine (UTM, INSTRON 3367, Instron, Norwood, MA, USA). It was performed with 30 kN of cell force and a test speed at 10 mm/min. The gauge length during the tensile test was fixed at 20 mm.

The oxygen permeability measurements of all sample films were recorded using an oxygen transmission rate analyzer (702, Mocon, Minneapolis, MN, USA) according to the ASTM 3985. The permeability tests were performed at 23 °C and was 0% in a relative humidity under 100% O_2_ atmosphere. Oxygen permeability (*P*) can be calculated with the following equation:

(2) $$P=J_{o}l/p$$

where *J_o_* is the steady state permeate flux, *p* is the oxygen pressure, and *l* is the film thickness [26].

Water contact angle measurement was performed by contact angle analyzer (SmartDrop, FEMTOFAB, Seongnam, South Korea) at 23 °C and with a relative humidity of 30%. Measurements were made by dropping 3-μL water droplets on the film surface at least five times.

## 3. Results and Discussion

### 3.1. Morphological Properties

**Figure 2:**
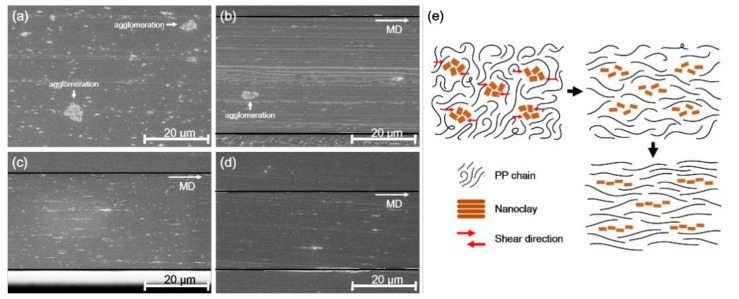
SEM micrographs of PP/nanoclay nanocomposite containing 2.0 wt% nanoclay according to the draw ratio: (**a**) ×1.0, (**b**) ×9.0, (**c**) ×13.5, (**d**) ×18.0, and (**e**) schematic presentation of overall morphology evolution for biaxially stretched PP/nanoclay nanocomposite.

Figure 2 illustrates the SEM images of the microtomed cross-section of the PP/nanoclay nanocomposite containing 2.0 wt% of nanoclay. As the draw ratio was increased from ×1.0 to ×18.0, the morphological change of the nanoclay in the PP matrix was clearly observed.

In Figure 2a, the nanoclay is dispersed in the PP matrix as the local agglomerations with a size of approximately 5 μm. However, it was observed that the agglomeration of the nanoclay gradually reduced as the draw ratio increased. In Figure 2c,d, with draw ratios of ×13.5 and ×18.0, this phenomenon was even more pronounced, and agglomeration of nanoclay was not found. This result can be attributed to the chain behavior of PP that occurs during the stretching of the PP/nanoclay nanocomposite. Once the randomly distributed PP chains are stretched, they are oriented according to the stretching direction. At this time, the nanoclay agglomerates are forced into shear stress by the PP chains, so that the agglomerates of the nanoclay are crushed or compressed. Another possibility is that the rupture mechanism may have an effect on the disintegration of agglomerates of nanoclays [27]. Since the PP/nanoclay nanocomposite undergoes the biaxial stretching process in the semi-molten state, it is assumed that the high viscos polymer transfers shear stress to the particle agglomeration and the rupture phenomenon occurs.

**Figure 3:**
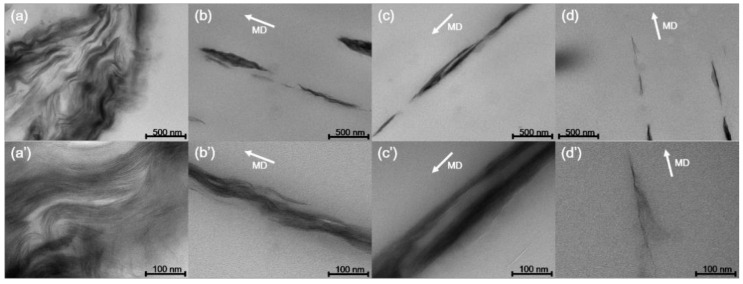
TEM images of PP/nanoclay nanocomposite containing 2.0 wt% nanoclay according to the draw ratio: (**a**), (**a’**) ×1.0; (**b**), (**b’**) ×9.0; (**c**), (**c’**) ×13.5; (**d**), (**d’**) ×18.0.

To observe the behavior of nanoclay according to the increase in draw ratio, the TEM image of the PP/nanoclay nanocomposite containing 2.0 wt% nanoclay is shown in Figure 3. As can be seen from Figure 3a,b, when the draw ratio increased from ×1.0 to ×9.0, the aggregated nanoclays were separated and more dispersed in the PP matrix. Further, it was confirmed that the plate-shaped nanoclay was exfoliated as the draw ratio was sequentially increased to ×13.5 and ×18.0. As observed from the SEM analysis, it was revealed that, from the TEM analysis, the nanoclay was uniformly dispersed in the PP matrix due to the shear stress exerted by the PP matrix when the PP/nanoclay nanocomposite film was exposed to sequential biaxial stretching. Note that, since these completely exfoliated nanoclays in the PP matrix were not observed under the low magnification of the SEM analysis, the amount of nanoclays shown in Figure 2d was smaller than that shown in Figure 2c.

### 3.2. Interlayer Distances Properties of Nanoclay

**Figure 4:**
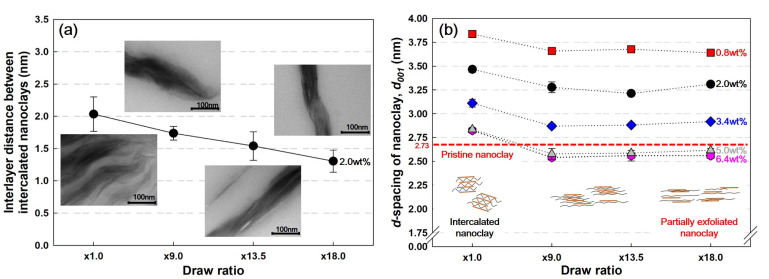
Nanoclay interlayer characteristics by (**a**) TEM and (**b**) XRD measurements (the number next to the plots in the graph is the content of nanoclay contained in the PP/nanoclay nanocomposite).

Figure 4a shows the interlayer distance of intercalated (nonexfoliated) nanoclays in PP/nanoclay nanocomposite containing 2.0 wt% of nanoclay using TEM analysis. The interlayer distance value of the intercalated nanoclay decreased from 2.03 to 1.30 nm during the increase in the draw ratio. The decrease in this value is a result from the biaxial stretching process because a stronger shear stress is applied to the nanoclay clusters in the PP matrix as the draw ratio increases. At this time, the nonexfoliated nanoclays are compressed by an external force, and the interlayer distance is considerably reduced.

Figure 4b shows the interlayer distance values of nanoclays depending on the variation of nanoclay content in PP/nanoclay nanocomposites. It was found that the nanoclay interlayer spacing decreases with the increase in nanoclay content. As the nanoclay content increased, nanoclay agglomeration increased, and the interlayer distance value decreased as the PP chains were too insufficient to effectively penetrate between the nanoclays [4].

From Figure 4a,b, it was found that there is a fairly significant difference in the interlayer distance values over the entire range of draw ratios for PP/nanoclay nanocomposite containing 2.0 wt% of nanoclay. The difference in these values was because the nanoclay interlayer distance values obtained using XRD analysis includes both intercalation and exfoliation cases. Figure 4b shows a different trend from the result of Figure 4a, illustrating that the d-spacing value of the nanoclay decreases in the range of ×1.0–×9.0 and then remains constant between the draw ratios of ×9.0 and ×18.0. From these results, it was certified that the amount of exfoliated nanoclay increased with the increase in the draw ratio of the PP/nanoclay nanocomposite, and the biaxial stretching process was found to be an effective process for the uniform dispersity of the nanoclay in the PP matrix.

### 3.3. Thermal Stability Properties

**Figure 5:**
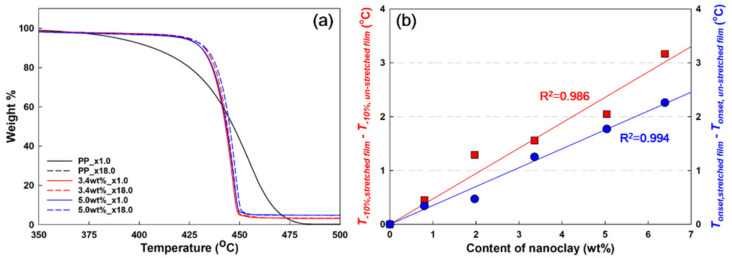
(**a**) Thermogravimetric (TG) curves and (**b**) difference in values (T_−10%_ and T_onset_) of unstretched films (×1.0) and stretched films (×18.0) of neat PP and PP/nanoclay nanocomposites.

It is known that the addition of nanoclay to PP improves the thermal stability due to the barrier effect that hinders the diffusion of volatile substances or gases [28,29]. TGA analysis was performed to compare the thermal stability of samples according to the nanoclay contents and dispersions in the PP/nanoclay nanocomposites. Referring to Figure 5a, it was clarified that PP starts pyrolysis at 388 °C, whereas PP/nanoclay nanocomposites containing 3.4 and 5.0 wt% of nanoclay, respectively, start pyrolysis at approximately 420 °C.

In the cases of the unstretched PP/nanoclay nanocomposite films, the thermogravimetric (TG) curves were almost similar, even though the amount of nanoclay was 1.6 wt% more, indicating that it did not contribute to the improvement of thermal stability with the increase in the nanoclay content. However, when stretching at a draw ratio of ×18, a different trend was observed, especially when the content of nanoclay in the PP matrix increased from 3.4 wt% to 5.0 wt%, wherein the TG curves showed significant differences. Based on the TG curves, Figure 5b shows the difference in decomposition temperatures (T_−10%_ and T_onset_) of the stretched and unstretched films of the PP/nanoclay nanocomposites. It was shown that the differences in decomposition temperatures increased linearly with the increase in nanoclay content. For that reason, the improvement in thermal stability can be attributed to the nanoclay content. These results serve as more evidence of the improvement in nanoclay dispersion in the PP matrix through the biaxial stretching process.

### 3.4. Mechanical Properties

**Figure 6:**
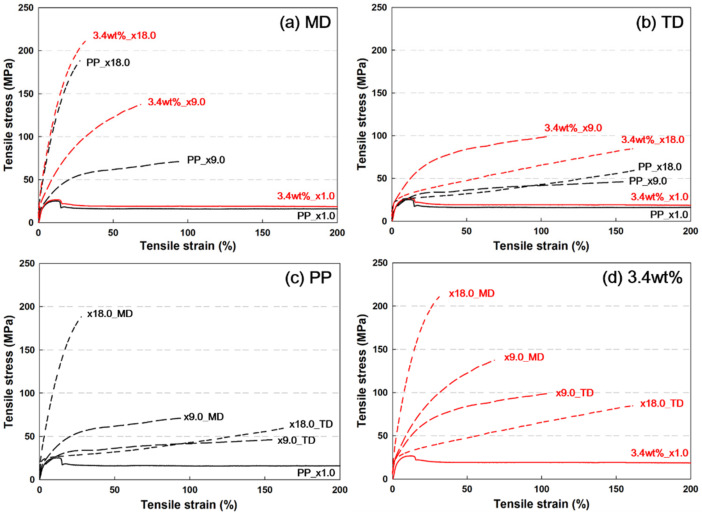
Stress–strain (s–s) curves in (**a**) MD and (**b**) TD of (**c**) PP and (**d**) PP/nanoclay nanocomposite containing 3.4 wt% nanoclay as a function of draw ratio.

Figure 6 shows the stress–strain (s–s) curves of PP and PP/nanoclay nanocomposites containing 3.4 wt% nanoclay according to the draw ratio. In Figure 6a,b, both PP and PP/nanoclay nanocomposites showed that the tensile strength and Young’s modulus increased and the elongation at break decreased with the increase in the draw ratio. These results indicate that the stiffness of PP and PP/nanoclay nanocomposites is strengthened due to the biaxial stretching process. Further, it was certified that the tensile strength and Young’s modulus were improved in the MD and TD of the PP/nanoclay nanocomposite containing 3.4 wt% nanoclay compared to PP. At a draw ratio of ×1.0 without stretching, the 3.4 wt% PP/nanoclay nanocomposite showed a slight increase in tensile strength compared with PP. However, it was found that the difference in tensile strength and Young’s modulus of PP and the PP/nanoclay nanocomposite increased as the draw ratio increased. In the MD, and at a draw ratio of ×18.0, the tensile strength and Young’s modulus of PP were 184.3 MPa and 1.63 GPa (Figure 6c), respectively, whereas the 3.4 wt% PP/nanoclay nanocomposite showed 210.1 MPa and 2.26 GPa (Figure 6d), respectively. The Young’s modulus of PP and the PP/nanoclay nanocomposite increased by approximately 116% and 190%, respectively, as the draw ratio increased from ×1.0 to ×18.0. In addition, at a draw ratio of ×18.0, the 3.6 wt% PP/nanoclay nanocomposite enhanced the Young’s modulus by about 39% compared to PP.

The s–s curves in the MD and TD showed different patterns, indicating that the tensile strength and Young’s modulus were relatively low in the TD. According to previous reports, in the case of sequential stretching, the strength and stiffness were higher in the second stretching direction, even if the stretching ratio was the same (3.0 × 3.0) [30]. More interestingly, the behavior of the s–s curve changes when the draw ratio increases from ×9.0 to ×18.0 in the TD. Both PP and PP/nanoclay nanocomposite exhibited the s–s curve behavior of elastomers or rubbery polymers at a draw ratio of ×18.0. This phenomenon is a result of the influence of molecular orientation. When the draw ratio is stretched in the MD from ×9.0 to ×18.0, the stacked lamellar crystal structure is deformed in a more ductile manner [30]. Therefore, as the draw ratio increased from ×9.0 to ×18.0, the yield stress of PP and PP/nanoclay nanocomposites decreased and the elongation at break increased.

**Figure 7:**
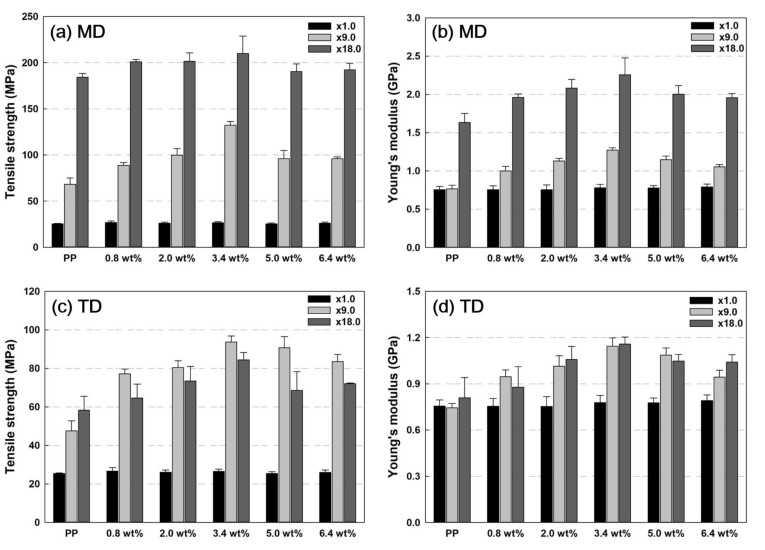
Tensile strength and Young’s modulus results according to MD and TD of PP and PP/nanoclay nanocomposites: Tensile strength in (**a**) MD and (**c**) TD and Young’s modulus in (**b**) MD and (**d**) TD.

Figure 7 shows the values of tensile strength and Young’s modulus according to the increase in the nanoclay content of the PP/nanoclay nanocomposite. In MD, when the nanoclay content in the PP matrix was increased, the tensile strength and Young’s modulus tended to increase with the increase in the draw ratio. In this study, the highest mechanical property performance was exerted when the content of nanoclay was 3.4 wt% in PP. Up to a content of 3.4 wt%, the nanoclay acted as a reinforcing agent for improving mechanical properties, but when the nanoclay content exceeded 5.0 wt%, the agglomeration of the nanoclay inhibited the tensile strength and Young’s modulus.

For TD, as seen in Figure 7c, the tensile strength tended to decrease when the draw ratio increased from ×9.0 to ×18.0 for all PP/nanoclay nanocomposites except neat PP. However, there are no significant differences between Young’s modulus of samples at different draw ratios for TD compared with MD. First, as mentioned earlier, it is presumed that these results appear as the ductility property occurs due to the change in the crystal structure as the draw ratio increases from ×9.0 to ×18.0. The second possibility is due to the morphology of nanoclays. In the SEM and TEM images of Figure 2 and Figure 3, it was observed that the nanoclays were arranged in the MD but acted as a defect when tensioned with TD.

### 3.5. Oxygen Barrier Properties

**Figure 8:**
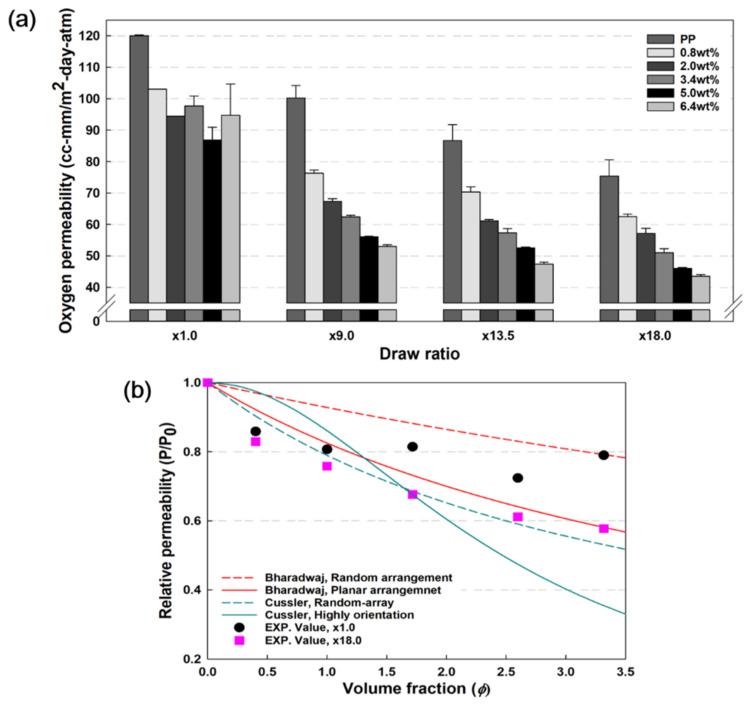
(**a**) Oxygen permeability results of PP and PP/nanoclay nanocomposites according to the increase in draw ration at RH 0% and (**b**) Relative permeability of PP/nanoclay nanocomposites with different content of nanoclay and draw ratio with different model predicted gas permeability.

Figure 8a presents the oxygen permeability results of PP and PP/nanoclay nanocomposites with respect to the draw ratio and nanoclay content. PP without biaxial stretching (×1.0) exhibited an oxygen permeability of 120.0 cc·mm/m^2^·day·atm. At this time, the oxygen permeability of PP decreased, and the oxygen barrier properties were improved with the increase in the draw ratio. At a draw ratio of ×18.0, PP showed a value of 75.4 cc·mm/m^2^·day·atm, which decreased by approximately 37%. At ×1.0, the oxygen permeability decreased with an increase in the nanoclay content in PP, but the decreasing trend and regularity disappeared when it reached 3.4 wt%. This is a result of the nanoclay not sufficiently forming tortuous pathways as it aggregates within the PP matrix.

However, after the biaxial stretching process of PP/nanoclay nanocomposites, the oxygen permeability decreased with an increase in nanoclay content. The 6.4 wt% PP/nanoclay nanocomposite showed an oxygen permeability value of 43.5 cc·mm/m^2^·day·atm, which was reduced by about 64% compared to PP at a draw ratio of ×18.0. There are several factors that affect oxygen barrier properties, such as molecular weight, crystallinity, molecular entanglement, and the internal structure of a polymer matrix. In this study, through biaxial stretching of PP/nanoclay nanocomposites, the oxygen barrier properties were enhanced due to the reduction of free volume inside the PP matrix and chain entanglement. In addition, as the biaxial stretching process affects the dispersion of the nanoclay, the formation of a tortuous path inside the PP matrix is one of the effective factors for improving the oxygen barrier properties. A synergistic effect was achieved due to the combination of these complex factors, resulting in improved oxygen barrier properties.

Furthermore, in order to evaluate the dispersion effect of nanoclay improved through the biaxial stretching process, the experimental value of oxygen permeability of PP/nanoclay nanocomposites were compared with the theoretical value. The theoretical relative permeability (*R_p_* = *P*/*P*_0_), according to the change in the content of nanoclay, was calculated through Bharadwaj [31] and Cussler models [32,33], and is summarized in Figure 8b. Moreover, the formulas for each model are shown in Table 1.

*P* and *P*_0_ are the oxygen permeability values of PP/nanclay nanocomposites and PP, respectively. *P*_0_ is applied separately for the oxygen permeability values of PP when the draw ratio is ×1.0 and ×18.0. *α* is the aspect ratio of the nanoclay, which was roughly estimated to be about 40 [34]; ∅ represents the volume fraction of nanoclay in the PP/nanoclay nanocomposite, calculated through the following equation:

(3) $$\phi =w\rho_{PP}/\left[w\rho_{PP}+\left(1-w\right)\rho_{nanoclay}\right]$$

where w is weight content of nanoclay. $\rho_{PP}$ and $\rho_{nanoclay}$ are the density of PP and nanoclay, which are selected as 0.89 g/cm^3^, 1.77 g/cm^3^, respectively.

Although the theoretically predicted values of the Bharadwaj and Cussler models are slightly different, in this work, we compared the experimental values with both of these models. At a draw ratio of ×18.0, the difference in R_p_ of PP/nanoclay nanocomposites was significantly different compared to ×1.0. This trend was even more pronounced when the content of nanoclay was 1.72 vol% (3.4 wt%) or more. It was shown that the biaxial stretching process acts as a factor to improve the arrangement regularity of nanoclay. The experimental values of R_p_ in this study, with a draw ratio of ×18.0, were shown to be similar to the theoretical values calculated by the Bharadwaj (planar arrangement) and Cussler (random-array) models. On the other hand, when the content of nanoclays exceeded 1.72 vol%, it was inconsistent with the theoretical values of the Cussler model, which predicted completely exfoliated and highly aligned morphology, as well as our experimental values. This is the result of slight agglomeration as the content of nanoclays increases, similar to the previous tensile strength and Young’s modulus results in Figure 7.

**Figure 9:**
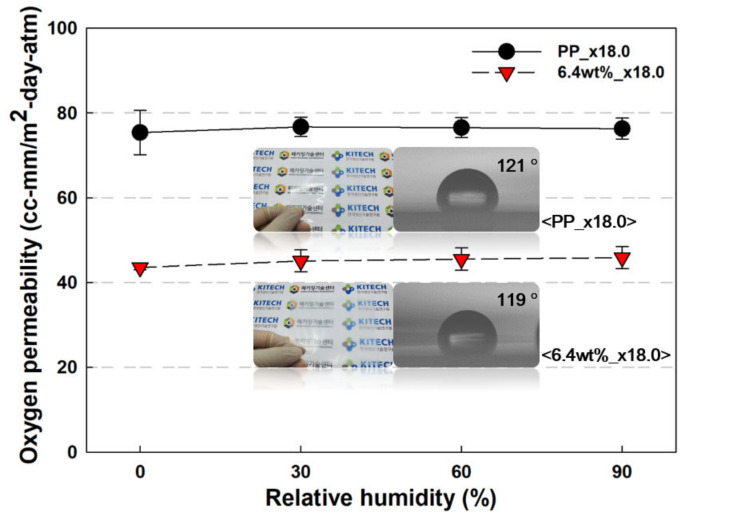
Oxygen permeability results of PP and PP/nanclay nanocomposite according to the increase in RH % (visual comparison of the samples and contact angle results are included in the graph).

Another important point regarding oxygen barrier properties is the maintenance of oxygen permeability during increasing relative humidity. This is because, if the oxygen barrier performance deteriorates due to the climate outside the packaging, the material, or the humidity inside the packaging material, the product will be adversely affected. The oxygen permeability of the samples was analyzed by increasing the relative humidity (RH), as shown in Figure 9. The 6.4 wt% PP/nanoclay nanocomposite with a draw ratio of ×18.0 showed the best oxygen barrier performance, and it maintained its performance even under extreme conditions of 90% RH. PP is a hydrophobic polymer, and as the nanoclay is located on the inside rather than the surface of PP, it allows PP to gain excellent water vapor barrier performance. The water contact angles of PP and 6.4 wt% PP/nanoclay nanocomposite with a draw ratio of ×18.0 were measured with similar values of 121° and 119°, respectively, indicating a hydrophobic surface. Due to the high contact angle, that is, the hydrophobic surface, it is difficult for moisture to be adsorbed by the film surface or diffused into the film, thereby realizing high moisture barrier properties [35]. Thus, even under high RH conditions, the oxygen permeability of the PP/nanoclay nanocomposite was maintained without deterioration.

## 4. Conclusions

In this study, PP/nanoclay nanocomposites were prepared by increasing the nanoclay content up to 6.4 wt%, and the mechanical and oxygen permeability changes of PP/nanoclay nanocomposites through biaxial stretching were evaluated. It was confirmed through morphological analyses that the nanoclays aggregated inside the PP matrix had improved dispersibility after the biaxial stretching process. In addition, partial exfoliation of nanoclays was also observed through XRD and TEM analysis. Since the biaxial stretching process has a positive effect on the dispersion of nanoclay in the PP matrix, its thermal, mechanical, and oxygen barrier properties were evaluated as being superior to those of conventional (unstretched) PP/nanoclay nanocomposites. It is worthwhile mentioning that this method does not require any chemical treatment such as toxic reagents or large amounts of solvents. Therefore, the biaxially stretched PP/nanoclay nanocomposite has excellent potential to be used as an ecofriendly packaging material. In addition, this study provided one representative approach for the dispersal of additives, including plate-like morphology, into polymers.

## Figures and Tables

**Table 1:** Models for predicting barrier properties of nanoclay filled nanocomposites.

Model	Formulas	Considered Parameters	Reference
Bharadwaj	$P/P_{0}=\left(1-\phi \right)/\left[1+\alpha /3\left(S+1/2\right)\phi \right]$	*S* = 0, Random arrangement	[31]
		*S* = 1, Planar arrangement	
Cussler	$P/P_{0}=\left(1-\phi \right)/{\left(1+2\alpha \phi /3\right)}^{2}$	Random-array	[32,33]
	$P/P_{0}={\left[1+\left\{\left(\alpha^{2}\phi^{2}\right)/\left(1-\phi \right)\right\}\right]}^{-1}$	Highly orientation	

## Data Availability

Not applicable.
